# HPMA-Based Copolymers Carrying STAT3 Inhibitor Cucurbitacin-D as Stimulus-Sensitive Nanomedicines for Oncotherapy

**DOI:** 10.3390/pharmaceutics13020179

**Published:** 2021-01-28

**Authors:** Marina R. Tavares, Klára Hrabánková, Rafał Konefał, Martin Kaňa, Blanka Říhová, Tomáš Etrych, Milada Šírová, Petr Chytil

**Affiliations:** 1 Institute of Macromolecular Chemistry of the Czech Academy of Sciences, Heyrovského náměstí 2, CZ-162 06 Prague 6, Czech Republic; tavares@imc.cas.cz (M.R.T.); konefal@imc.cas.cz (R.K.); etrych@imc.cas.cz (T.E.); 2 Institute of Microbiology of the Czech Academy of Sciences, Vídeňská 1083, CZ-142 20 Prague 4, Czech Republic; klara.hrabankova@biomed.cas.cz (K.H.); martin.kana@biomed.cas.cz (M.K.); rihova@biomed.cas.cz (B.Ř.); sirova@biomed.cas.cz (M.Š.)

**Keywords:** cucurbitacin-D, immuno-oncotherapy, *N*-(2-hydroxypropyl) methacrylamide (HPMA), pH-controlled release, signal transducer and activator of transcription 3 (STAT3) inhibition

## Abstract

The study describes the synthesis, physicochemical properties, and biological evaluation of polymer therapeutics based on *N*-(2-hydroxypropyl)methacrylamide (HPMA) copolymers intended for a tumor-targeted immuno-oncotherapy. Water-soluble linear and cholesterol-containing HPMA precursors were synthesized using controlled reversible addition–fragmentation chain transfer polymerization to reach molecular weight *M*_n_ about 2 × 10^4^ g·mol^−1^ and low dispersity. These linear or self-assembled micellar conjugates, containing immunomodulatory agent cucurbitacin-D (CuD) or the anticancer drug doxorubicin (Dox) covalently bound by the hydrolytically degradable hydrazone bond, showed a hydrodynamic size of 10–30 nm in aqueous solutions. The CuD-containing conjugates were stable in conditions mimicking blood. Importantly, a massive release of active CuD in buffer mimicking the acidic tumor environment was observed. In vitro, both the linear (LP-CuD) and the micellar (MP-CuD) conjugates carrying CuD showed cytostatic/cytotoxic activity against several cancer cell lines. In a murine metastatic and difficult-to-treat 4T1 mammary carcinoma, only LP-CuD showed an anticancer effect. Indeed, the co-treatment with Dox-containing micellar polymer conjugate and LP-CuD showed potentiation of the anticancer effect. The results indicate that the binding of CuD, characterized by prominent hydrophobic nature and low bioavailability, to the polymer carrier allows a safe and effective delivery. Therefore, the conjugate could serve as a potential component of immuno-oncotherapy schemes within the next preclinical evaluation.

## 1. Introduction

Conventional cancer chemotherapy often brings unsatisfactory results and/or significant toxicity; thus, new treatment alternatives to defeat cancer are demanded [1,2]. There is now strong evidence documented in the literature to support the application of immunotherapeutic protocols for the treatment of cancer including reactivation of the immune system against cancer cells to suppress the tumor growth. Researchers have become increasingly interested in immuno-oncotherapy, e.g., the use of immune checkpoint inhibitors, since a large body of data showing the power of this strategy has been reported [1,2,3,4,5,6]. Even though the use of checkpoint inhibitors has generated promising results, solo immunotherapy is still limited. Therefore, research groups have been exploring the synergic effect resulted from the combination of immunotherapy with chemotherapy.Combination therapies have shown higher therapeutic efficacies, suggesting that this strategy may achieve promising outcomes for the cancer treatment scenario [2,4,5,7,8].

The signal transducer and activator of transcription 3 (STAT3) is an oncogenic signaling protein expressed in a variety of tumor type cells, as well as in immune cells in the tumor environment, emerging as an important molecule for immuno-oncotherapy. In addition to lymphomas and leukemia, the pathological activation of STAT3 was also found in several solid tumors, such as prostate, pancreatic, lung, breast, ovarian, head and neck cancers [9]. The activation of the STAT3 pathway supports tumor angiogenesis, growth, invasion, tumor cell proliferation and survival, in addition to facilitating evasion from the immune surveillance; therefore, this molecule is a promising target for cancer therapy [6,9,10].

The inhibition of STAT3 activity also increases the antitumor T cell-mediated immune response, leading to apoptosis of tumor cells and inhibition of tumor growth or even tumor regression [6,9]. Moreover, diverse STAT3 inhibitors have already been shown to be effective by inducing the apoptosis of tumor cells [10,11,12,13,14]. Cucurbitacins are highly oxygenated tetracyclic triterpenes, which show potent pharmacological effects, including anticancer activity [15,16,17,18,19,20]. Moreover, the dose necessary for cucurbitacins to present a therapeutical effect is much lower than the toxic dose, thus increasing their potential as therapeutic agents [19]. Among cucurbitacin derivatives, cucurbitacin-D (CuD) showed an effective inhibitory effect on the proliferation of colon, breast, lung, cervical, and prostate cancer cell lines as a result of Janus kinase/signal transducer activator of transcription 3 (JAK/STAT3) signaling pathway inhibition [15,16,18,19,20]. Studies also identified that the combination of cucurbitacins and anticancer agents can promote a synergism and may augment the chemotherapeutic effects via suppression of STAT3, being promising for tumor treatment [17,20].

However, there are significant limitations concerning the potential clinical application of cucurbitacins due to their poor water solubility [21], high toxicity, low selectivity, and narrow therapeutic window for treatment [15,17]. To overcome these limitations, polymeric carriers were designed for effective solubilization of cucurbitacins, controlled delivery, and sustained rate of release in cancerous tissue. Micellar formulations were able to deliver cucurbitacin to tumor cells and inhibit STAT3 [21]. Moreover, polymer materials used as nanocarriers of bioactive compounds are passively accumulated in solid tumors as a result of the enhanced permeability and retention (EPR) effect [22,23]. When compared to smaller molecules, biomaterials with increased hydrodynamic size can accumulate in the tumor tissue at a higher extent [24] and those bigger than 10 nm, thus exceeding the renal filtration limit, can reach circulation several times longer than free parent drugs [25]. This effect contributes to the improvement of pharmacokinetic profile and therapeutic efficacy when compared to the conventional anticancer treatment with low-molecular-weight therapeutics [22,26,27]. Indeed, the covalent bonding or encapsulation of low-molecular-weight drugs significantly reduces the side effects of carried drugs as the drug activity is generally “switched off” when bound to the carrier. This is an advantage concerning the safety of the treatment when potent immunomodulatory agents are used since these molecules are associated with a high incidence of immune-related adverse events [5].

For the design of an ideal macromolecular antitumor “prodrug” conjugate, the covalent bond between the drug and the polymer backbone must be relatively stable in the blood to prevent the drug release during its transport and activation at the appropriate site of its desired pharmacological effect, i.e., in the tumor tissue. As a result of hypoxic conditions in solid tumors, the typically lower pH of tumor environment induces pH-dependent hydrolysis; thus, the anticancer drugs can advantageously be linked to the polymer backbone via pH-sensitive hydrolytically degradable bonds. In that case, drug release from the carrier is triggered through lower pH, i.e., the pH of the tumor environment (pH ≈ 6.5) and the endocytic vesicle (pH ≈ 5–6) or secondary lysosomes (pH ≈ 5), in which the biomaterials localize after internalization into tumor cells, is lower than the physiological pH of circulation (pH ≈ 7.4) [24,28,29,30].

There have been many studies describing the characteristics of polymer carriers based on *N*-(2-hydroxypropyl) methacrylamide (HPMA), which are water-soluble, biocompatible, nonimmunogenic, and nontoxic. They are known to prolong the circulation time of the therapeutics, i.e., drugs and/or ligands, deliver them to their biological sites, i.e., tissues or receptors, increase the tumor accumulation, and reduce adverse effects of therapy. HPMA copolymers bearing various cytostatic drugs have been extensively tested and proved excellent antitumor activity in numerous mouse and human tumors [31,32]. Recently, an HPMA copolymer–pirarubicin conjugate showed important anticancer efficacy during “compassionate use” in humans [33]. Several polymer–drug conjugates were introduced into clinical trials [30]. The limited clinical approval of HPMA polymer-based nanomedicines is based mainly on their rather complicated synthesis, unsatisfactory characteristics, and tendency of such polymer systems to perform differently in preclinical animal models and humans. Nevertheless, HPMA polymer-based nanomedicines called SDX-7320 developed by SynDevRx Inc. recently entered clinical trials. Results of the phase 1 dose-escalation study showed promising results in slowing down the rate of disease progression and formation of new metastases [34].

There is now much evidence concerning the use of pH-sensitive spacers for binding various cytotoxic anticancer drugs to HPMA polymer carriers, i.e., using the hydrazone bond susceptible to hydrolysis in a mildly acidic environment [28,30]. Studies presented the synthesis of conjugates based on HPMA copolymers in which anticancer drugs, such as doxorubicin (Dox), pirarubicin, or docetaxel, were conjugated via a pH-sensitive hydrazone bond showing high potential for an effective in vivo anticancer activity. Furthermore, the advantageous combination of hydrophilic polymers with highly hydrophobic drugs enables the design and development of self-assembled micellar carrier systems with advanced pharmacokinetic and antineoplastic activity [30]. Indeed, auxiliary hydrophobic moieties, such as cholesterol or its derivatives, can be introduced to HPMA copolymers, thus forming amphiphilic copolymers, which self-assemble into micelles in aqueous solutions, increasing their size and molecular weight [25,35,36,37,38]. Additionally, the synthesis, as well as the physicochemical and biological evaluation, of several drug delivery systems containing immunomodulators, including all-trans-retinoic acid bound to HPMA copolymer, aimed at improving tumor immunotherapy, was recently documented. [39]

In this paper, we provide the design, synthesis, and characterization of linear and micellar HPMA precursors and the respective conjugates bearing the immunomodulatory agent CuD bound by the pH-sensitive hydrazone bond, as well as the preliminary biological evaluation. Importantly, the combined in vivo anticancer efficacy of developed CuD-containing nanomedicines with micellar HPMA copolymers containing the anticancer drug Dox bound by the hydrazone bond is presented and discussed as a promising strategy for advanced cancer treatment. The combination of immunotherapy and oncotherapy should promote the immunomodulatory intervention by STAT3 inhibition, improving the outcome of the disease.

## 2. Experimental Section

### 2.1. Materials

2,2′-Azobisisobutyronitrile (AIBN), 2-cyanopropan-2-yl dithiobenzoate (CTA-AIBN), 2,4,6-trinitrobenzene-1-sulfonic acid (TNBSA), methacryloyl chloride, Nile red (NR) (BioReagent, ≥ 98%), *N*,*N*-dimethylacetamide (DMA), and *tert*-butyl alcohol were purchased from Sigma-Aldrich (Prague, Czech Republic). Cucurbitacin-D (CuD) was from Extrasynthese (Genay, France) and 2,2′-azobis(4-methoxy-2,4-dimethylvaleronitrile) (V-70) was from Fujifilm Wako Chemicals Europe (Neuss, Germany). All other solvents and chemicals were of analytical grade.

### 2.2. Synthesis of Monomers and Chain Transfer Agent

The monomer HPMA was synthesized by the reaction of methacryloyl chloride and 1-aminopropan-2-ol in CH_2_Cl_2_ in the presence of anhydrous sodium carbonate as described previously. The monomer cholest-5en-3β-yl 6-methacrylamido hexanoate (MA-AH-cholesterol) was synthesized by the reaction of MA-AH-OH and cholesterol in tetrahydrofuran (THF) in the presence of *N*,*N*’-dicyclohexylcarbodiimide (DDC) and 4-(dimethylamine)pyridine. The monomer *N*-(*tert*-butoxycarbonyl)-*N*’-(6-methacrylamidohexanoyl)hydrazine (MA-AH-NHNH-Boc) was prepared in two-step synthesis. First, *N*-methacryloyl-6- aminohexanoic acid was prepared then reacted with *tert*-butyl carbazate in THF [35,40,41].

The chain transfer agent S-2-cyano-2-propyl-S′-ethyl trithiocarbonate (CTA-TTC) was synthesized as described before [42]. The purity of monomers and CTA-TTC was examined by high-performance liquid chromatography (HPLC) analysis using a Shimadzu HPLC system with a C18 reversed-phase Chromolith Performance RP-18e column and a diode array detector (Shimadzu SPD-M20A, Shimadzu, Tokyo, Japan). The eluent consisted of water/acetonitrile and a gradient of 5–95% *v*/*v* acetonitrile was applied, using 5 mL·min^−1^ as a flow rate.

### 2.3. Synthesis of Polymer Precursors

#### 2.3.1. Linear Polymer Precursor LP

A statistical copolymer precursor composed of HPMA and a comonomer bearing Boc-protected hydrazide groups (MA-AH-NHNH-Boc) was prepared via controlled radical RAFT (reversible addition–fragmentation chain transfer) copolymerization, using CTA-TTC and the initiator V-70. The molar ratio of monomer/CTA-TTC/AIBN was 320/2/1 and the molar ratio of monomers HPMA/MA-AH-NHNH-Boc was 92/8. The polymerization was carried out in a mixture of *t*-butanol/DMA (85/15) at 30 °C for 72 h; then, the trithiocarbonate end group and Boc group were removed, respectively, via reaction with an excess of AIBN, thermally in distilled water, as previously described [43,44]. The detailed synthetic procedure is shown in the Supplementary Materials, Section 1.

#### 2.3.2. Micellar Polymer Precursor MP

A statistical copolymer precursor composed of HPMA, comonomers bearing Boc-protected hydrazide groups (MA-AH-NHNH-Boc), and hydrophobic cholesterol moiety (MA-AH-cholesterol) was prepared via controlled radical RAFT copolymerization, using CTA-AIBN and the initiator AIBN. The molar ratio of monomer/CTA-AIBN/AIBN was 350/2/1 and the molar ratio of monomers HPMA/MA-AH-NHNH-Boc/MA-AH-cholesterol was 89/9/2 (5 mol.% of hydrazide groups and 2.1 mol.% of cholesterol moieties). The polymerization was carried out in *t*-butanol at 70 °C for 16 h according to [35,38]. The dithiobenzoate end group and Boc group were removed as described above [43,44]. The detailed synthetic procedure is shown in the Supplementary Materials, Section 1.

### 2.4. Synthesis of Polymer Conjugates

#### 2.4.1. Polymer Conjugates Bearing CuD

Polymer–drug conjugates LP-CuD and MP-CuD were prepared via the reaction of hydrazide groups of LP or MP, respectively, with CuD in methanol in the dark at 10 °C. For LP-CuD, the polymer precursor LP (150 mg, 49.84 µmol hydrazide groups) was dissolved in 1.2 mL of dry methanol and 180 µL of acetic acid, and then slowly dropped into CuD solution (17.4 mg, 33.67 µmol) in 500 µL of dry methanol under stirring (750 RPM) at 10 °C. The reaction was carried out overnight in the dark. The polymer–drug conjugate LP-CuD was isolated by precipitation into ethyl acetate and dried under vacuum (133 mg; 79%).

The synthetic procedure for the micellar polymer–drug conjugate MP-CuD was analogous to the linear one (LP-CuD). The reaction of polymer MP (150 mg, 47.79 µmol of hydrazide groups) and CuD (15.2 mg, 29.48 µmol) in dry methanol (1.9 mL) and acetic acid (180 µL) afforded the conjugate MP-CuD (128.5 mg; 78%). NMR data for both conjugates and the neat CuD are shown in the Supplementary Materials, Section 2. For both conjugates LP-CuD and MP-CuD, 10.7 wt.% of CuD was added into the reaction mixture, and the final contents were 6.2 wt.% and 6.7 wt.%, respectively.

#### 2.4.2. Polymer Conjugates Bearing Dox

The polymer–drug conjugate (MP-Dox) of amphiphilic HPMA copolymer bearing cholest-4-en-3-one (2 mol.%) and doxorubicin (8.1 wt.%) bound by hydrazone bond was synthesized in methanol in the presence of acetic acid at room temperature overnight in the dark. The detailed synthesis and characterization were described previously [36,38].

### 2.5. Size-Exclusion Chromatography

A Shimadzu HPLC system equipped with size-exclusion chromatography (SEC) columns was used to determine the number-average molecular weight (*M*_n_), the weight-average molecular weight (*M*_w_), and the dispersity (*Ð*) of polymer precursors and polymer–drug conjugates. SPD-M20A photodiode array (Shimadzu, Tokyo, Japan), DAWN HELEOS II multiangle light scattering (MALS), and Optilab-rEX differential refractive index (RI) (both Wyatt Technology Co., Santa Barbara, CA, USA) detectors were used. The analysis was performed using a combination of TSKgel3000AW and TSKgel4000AW columns at a flow rate of 0.6 mL·min^−1^ and the mobile phase consisted of 80% of methanol and 20% of 0.3 M sodium acetate buffer, pH 6.5. ASTRA VI software was used for the calculation of *M*_w_, *M*_n_, and *Ð*. The Optilab-rEX detector enables the direct determination of sample *dn*/*dc*, and the solvent refractive index provides 100% recovery of the injected sample from the column.

### 2.6. Ultraviolet–Visible Light (UV–Vis) Spectrophotometry

The content of hydrazide-terminated side chains of the polymer precursors was determined by UV–Vis spectrophotometry using Specord 205 ST, Analytic Jena AG,( Jena, Germany). A modified TNBSA assay method was applied as described previously [45]. LP was dissolved in borate buffer (0.1 M Na_2_B_4_O_7_·10H_2_O, pH 9.3) at the concentration of 2 mg·mL^−1^. Then, 100 μL of this solution was mixed with borate buffer (875 μL) and a 0.03 M solution of TNBSA in water (25 μL), directly in the cuvette (l = 1 cm). After 90 min incubation, the absorbance was measured and the molar absorption coefficient used was ε = 17,200 L∙mol^−1^∙cm^−1^ (λ_max_ = 500 nm). For MP, the procedure for determination was analogous; however, the sample containing the cholesterol moiety was dissolved in borate buffer/dimethyl sulfoxide (DMSO) (9/1) [35]. The amount of Dox in the conjugate was determined spectrophotometrically using ε_488_ = 9800 L∙mol^−1^∙cm^−1^ in water (λ_max_ = 488 nm).

### 2.7. Nuclear Magnetic Resonance Spectrometry

^1^H-NMR of polymer precursors and polymer–drug conjugates was performed in a Bruker Avance III 600 spectrometer (Bruker, Billerica, MA, USA) operating at 600.2 MHz using DMSO-*d*_6_ as solvent. The NMR spectra of all samples were measured in 5 mm NMR tubes and the typical conditions for measurements of the spectra were as follows: π/2 pulse width 10 µs, relaxation delay 10 s, spectral width 10 kHz, acquisition time 3.21 s, and 200 scans. For the calculation of the cholesterol content, the integral intensities of *δ* ≈ 4.71 ppm (1 H, O*H*) and *δ* ≈ 3.67 ppm (1 H, C*H*) from the HPMA monomer unit were employed, and the content of cholesterol statistically distributed through the polymer precursor chain was determined using the signal with the integral intensity of *δ* ≈ 5.34 ppm (1 H, C*H* from c-6 of cholesterol moiety). For determination of the content of CuD attached to the polymer precursor, the integral intensities of *δ* ≈ 7.18 ppm (1 H, N*H*), *δ* ≈ 4.71 ppm (1 H, O*H*), and *δ* ≈ 3.67 ppm (1 H, C*H*) from the HPMA monomer unit were used, and the content of drug in the polymer–drug conjugates was assessed by using the signals with the integral intensities of *δ* ≈ 6.75 ppm (1 H, C*H* from c-23 of CuD) and *δ* ≈ 5.74 ppm (1 H, C*H* from c-6 of CuD). ^13^C-NMR of polymer–drug conjugates was recorded in a Bruker Avance III 600 spectrometer operating at 150.95 MHz using DMSO-*d*_6_ as solvent. The NMR spectrum was measured in 5 mm NMR tubes with the following measurement conditions: π/2 pulse width 8 µs, relaxation delay 10 s, spectral width 37 kHz, acquisition time 0.86 s, and 30,000 scans. The evaluation of which keto group reacted to form the hydrazone bond in the polymer–drug conjugate was assessed by using the signals with the integral intensities of *δ* ≈ 213 ppm (from c-11 of CuD), *δ* ≈ 212 ppm (from c-3 of CuD), and *δ* ≈ 204 ppm (from c-22 of CuD). In the case of MP-Dox, the content of cholest-4-en-3-one was calculated by ^1^H-NMR (600.2 MHz) as shown before [36].

### 2.8. Dynamic Light Scattering

Dynamic light scattering was performed to evaluate the hydrodynamic diameter (*D*_h_) of copolymer precursors and polymer–drug conjugates using a Nano-ZS instrument (ZEN3600, Malvern Panalytical, Malvern, UK), in which the intensity of the scattered light was detected at angle *θ* = 173° with a laser wavelength of 632.8 nm. The samples were dissolved in water (0.5 mg·mL^−1^) and filtered through a 0.45 µm polyvinylidene fluoride (PVDF) filter. The values were determined as a mean of at least three independent measurements.

### 2.9. Critical Micellar Concentration Determination

The critical micellar concentration (CMC) was determined by fluorescence using the dye Nile Red (NR) according to the literature [46,47]. The polymer precursor MP was dissolved in phosphate-buffered saline (PBS; 10 mM, pH 7.4) at a concentration of 1 mg·mL^−1^; then, 15 different dilutions (from 1000 to 0.2 mg·L^−1^) were prepared (the total volume of each dilution was 250 µL). A solution of NR in ethanol (6.37 mg·L^−1^) was prepared, and 12.5 µL of this solution was added to each sample dilution (the final concentration of NR in samples was 1 µM). The dilutions were prepared in duplicate and the fluorescence was measured at λ_ex_ = 550 and λ_em_ = 630 nm on a Synergy H1 hybrid Reader instrument (Biotek, Winooski, VT, USA) using a 96-well flat-bottom black microplate. The dependence of fluorescent intensity on the negative logarithm of the sample concentration was plotted, and CMC was determined by the intersection of two tangent lines drawn through points at lower and higher concentrations.

### 2.10. In Vitro Release of CuD from Linear Polymer–Drug Conjugate

CuD release was investigated at 37 °C in buffer solutions at pH = 7.4, modeling the blood conditions, and pH = 5.0, corresponding to the lysosome environment, in which the polymer conjugates are localized after internationalization into tumor cells. The rate of CuD release from LP-CuD was monitored by incubation of the sample in 0.1 M phosphate buffer with 0.05 M NaCl at pH 5.0 and 7.4 at 37 °C in the dark. Aliquots were collected at intervals from 0 to 30 h and the released drug was extracted into chloroform. The released amount of CuD was investigated by HPLC analysis using a Shimadzu HPLC system with a C18 reversed-phase Chromolith Performance RP-18e column and a diode array detector (Shimadzu SPD-M20A, Tokyo, Japan). The eluent consisted of water/acetonitrile, and a gradient of 5–95% *v*/*v* acetonitrile was applied, using 5 mL·min^−1^ as the flow rate. The relative area of peaks was used for the calculation, and all drug release data are expressed as the amount of free CuD relative to the total CuD content in the conjugate. All experiments were carried out in triplicate.

## 3. Biological Evaluation

### 3.1. In Vitro Cytotoxicity

The following murine and human tumor cell lines were purchased from ATCC (Manassas, VA, USA) and used for the in vitro evaluation of the cytotoxic/cytostatic activity of the conjugates containing CuD: murine EL4 T-cell lymphoma (ATCC TIB-39), 4T1 mammary carcinoma (ATCC CRL-2539), and CT26 colon adenocarcinoma (ATCC CRL-2638), and human ovarian carcinomas SK-OV-3 (ATCC HTB-77) and OVCAR-3 (ATCC HTB 161). Human prostate cancer cells DU145 were kindly provided by Dr. Z. Hodný from the Inst. of Molecular Genetics of the CAS, Prague, Czech Republic. The cells were maintained as recommended by the provider. The culture media and supplements were from Sigma-Aldrich (Prague, Czech Republic), whereas the fetal calf serum (FCS) was from Gibco (Paisley, UK). All cell lines were free of mycoplasma contamination (MycoAlert Mycoplasma Detection Kit, Lonza, Basel, Switzerland).

For the cytostatic effect, the cells were washed and seeded in 96-well flat-bottom tissue plates (Nunc, Thermo Fisher Scientific, MA, USA) in the culture medium at 5000 cells per well, whereas 4T1 cells were seeded at 2500 cells per well. The conjugates and free CuD were added in titrated concentrations. Next, the cells were cultivated for 72 h at standard conditions. For the last 6 h, ^3^H-thymidine was added at concentration 0.4 µCi·mL^−1^, and, at the end, the plates were frozen at −20 ℃. The final processing was done using a Tomtec Mach III harvestor, and the radioactivity was measured on the filter plate with a solid Meltilex scintillator using the Microbeta Trilux beta counter (Perkin Elmer, MA, USA). Alternatively, in the EL4 T-cell lymphoma cell line, the cytotoxic effect of the conjugates or CuD was evaluated using the standard 3-(4,5-dimethylthiazol-2-yl)-2,5-diphenyltetrazolium bromide (MTT) assay, as these cells do not incorporate thymidine at a sufficient quantity. The cytotoxicity toward normal cells was checked in murine spleen cells. They were seeded at a density of 100,000 per well with 5 µg·mL^−1^ concanavalin A (Sigma-Aldrich, Prague, Czech Republic) for polyclonal stimulation. Again, the cells were cultivated for 72 h, and MTT was used to determine the cytotoxic effect. The in vitro cytostatic/cytotoxic effects were expressed as half maximal inhibitory concentration (IC_50_) value, i.e., the concentration of cytotoxic drug that inhibits the proliferation or metabolic activity by 50%. At least four parallel samples were used for each experimental condition. Free CuD (Tocris Bioscience, Bristol, UK) was used as a control, dissolved in DMSO at 4 mM concentration, and further diluted in the culture medium.

### 3.2. In Vivo Antitumor Activity

Female BALB/c mice (H-2d) were obtained from the Inst. of Physiology of the CAS, Prague, Czech Republic and bred under conventional conditions. The 4T1 mammary carcinoma cells (ATCC CRL-2539) were injected subcutaneously (s.c.) with 2 × 10^5^ cells in 0.1 mL. Next, the mice were treated after tumors developed to a measurable size (diameter of the tumor focus 6–7 mm), i.e., at day 8 post tumor transplantation. The mice were treated either with the CuD-containing conjugate as a sole treatment or using a combination treatment with MP-Dox. Tumor size and survival were regularly monitored. Moreover, body weight and signs of systemic toxicity were scored following the treatment. For the toxicity studies, a body weight reduction of more than 15% was considered the cutoff value. Analysis of significance was conducted using Student’s t-test, and survival data were analyzed by log-rank (Mantel–Cox) test in Graph-Pad Prism software. The experiments were carried out in accordance with Council of Europe Convention European Treaty Series (ETS) 123 on the Protection of Vertebrate Animals used for Experimental and Other Scientific Purposes, and the Czech National Council Act 246/1992 on the protection of animals against cruelty, amended, and European Directive 210/63/EU on the protection of animals used in scientific research. The study was approved by the Laboratory Animal Care and Use Committee of the Institute of Microbiology of the Czech Academy of Sciences and the Department Committee of the Czech Academy of Sciences (Approval ID 107/2016, issued on 4.1.2017.). All efforts were made to minimize the number of animals used and to minimize suffering.

## 4. Results and Discussion

Here, we present the design, synthesis, and physicochemical and biological evaluation of the HPMA copolymer conjugates with STAT3 inhibitor CuD bound to the polymer via the pH-sensitive spacer designed for an effective tumor-targeting drug delivery and immuno-oncotherapy by a blockade of STAT3 signaling pathway in tumor cells. Moreover, the potential of combination therapy with previously developed anticancer nanomedicine containing drug Dox is described.

### 4.1. Synthesis and Physicochemical Properties of Copolymer Precursors and Polymer–Drug Conjugates

**Scheme 1:**
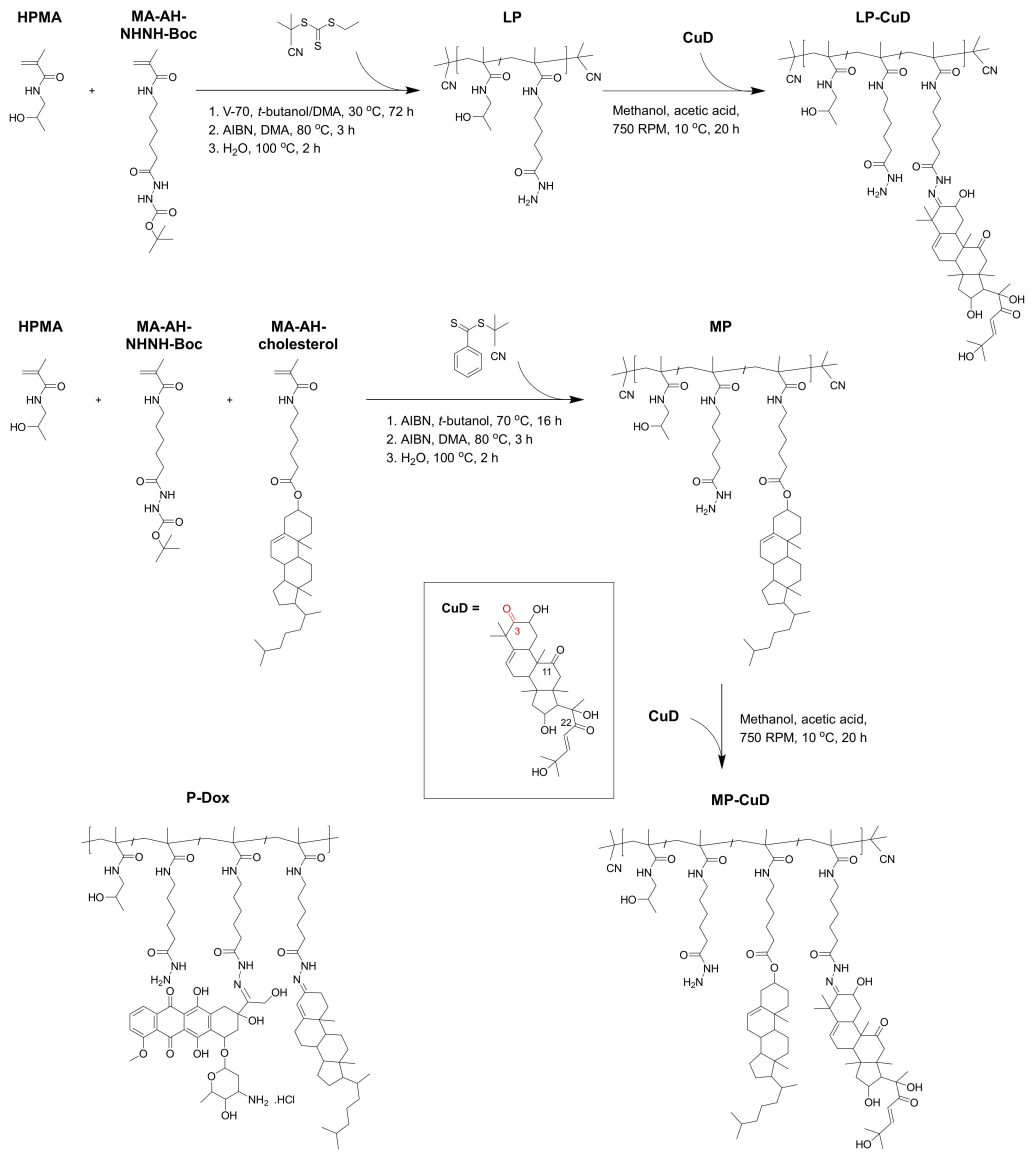
Synthesis of linear (LP) and micellar (MP) polymer precursors, as well as the respective polymer–drug conjugates LP-cucurbitacin-D (CuD) and MP-CuD. Structures of conjugate MP-doxorubicin (Dox) and cucurbitacin-D are also shown.

The controlled RAFT polymerization technique was employed to synthesize the polymer precursors LP and MP with molecular weight *M*_n_ of about 2 × 10^4^ g·mol^−1^ and a narrow dispersity (up to 1.2). Unlike the polymer precursor LP, the copolymer MP contained 2 mol.% covalently bound cholesterol substituents (see ^1^H-NMR spectrum of polymer precursor MP in the Supplementary Materials, Section 2, Figure S1), which was previously found to be the optimal composition for the self-assembly of this amphiphilic copolymer into micellar nanostructures in aqueous solutions [37]. Our previous studies [35,37,38] on micelle-forming amphiphilic HPMA copolymers with cholesterol derivatives showed polymer carriers with the mentioned *M*_w_ as suitable micellar drug carriers; thus, the previous conditions used for their synthesis were applied in this study. Polymer precursors LP and MP contained 4 and 5 mol.%, respectively, of hydrazide groups statistically distributed along the polymer chain, which were used for the consequent attachment of CuD or Dox via a hydrazone bond. Keeping the reaction in the cold highly suppressed a possible crosslinking between hydrazide groups of the polymer backbone and keto groups present in the CuD structure. The reaction scheme and studied polymer structures are shown in Scheme 1 and their physicochemical characteristics are described in Table 1. The CuD attachment was indicated by the appearance of hydrogen signals from carbon 6 and carbon 23 of CuD (see ^1^H-NMR spectra of conjugates LP-CuD and MP-CuD in the Supplementary Materials, Section 2, Figures S2 and S3, respectively). ^13^C-NMR was employed to evaluate which keto group from CuD was involved in the reaction with hydrazide groups of polymer precursor. The predominant interaction of the keto group from carbon 3 of CuD was proven by the disappearance of the carbon 3 peak together with the maintenance of peaks from carbons 11 and 22 of CuD in ^13^C-NMR spectra of the conjugate LP-CuD (see ^13^C-NMR spectra of conjugate LP-CuD and neat CuD in the Supplementary Materials, Section 2, Figure S4). The attachment of CuD to the polymer carriers by the pH-sensitive hydrazone bond slightly increased the values of molecular weight *M*_w_ and dispersity. Nevertheless, both polymer–CuD conjugates showed suitable molecular characteristics for the consequent physicochemical and biological evaluation.

In the case of the micellar polymer–Dox conjugate (MP-Dox), the polymer precursor containing hydrazide groups (5 mol.%) was used for consequent reaction with the keto group of both cholest-4-en-3-one and Dox (see structure in Scheme 1). The synthetic procedure and in vivo behavior evaluation of micellar conjugate with Dox bound via a pH-sensitive hydrazone bond was described earlier [25,36]. The hydrodynamic size (*D*_h_) of micelles was found to be approximately 20–30 nm, exceeding the size of linear samples (*D*_h_ ≈ 10 nm); therefore, we expected a higher accumulation of micellar conjugates in solid tumors due to the EPR effect.

**Figure 1:**
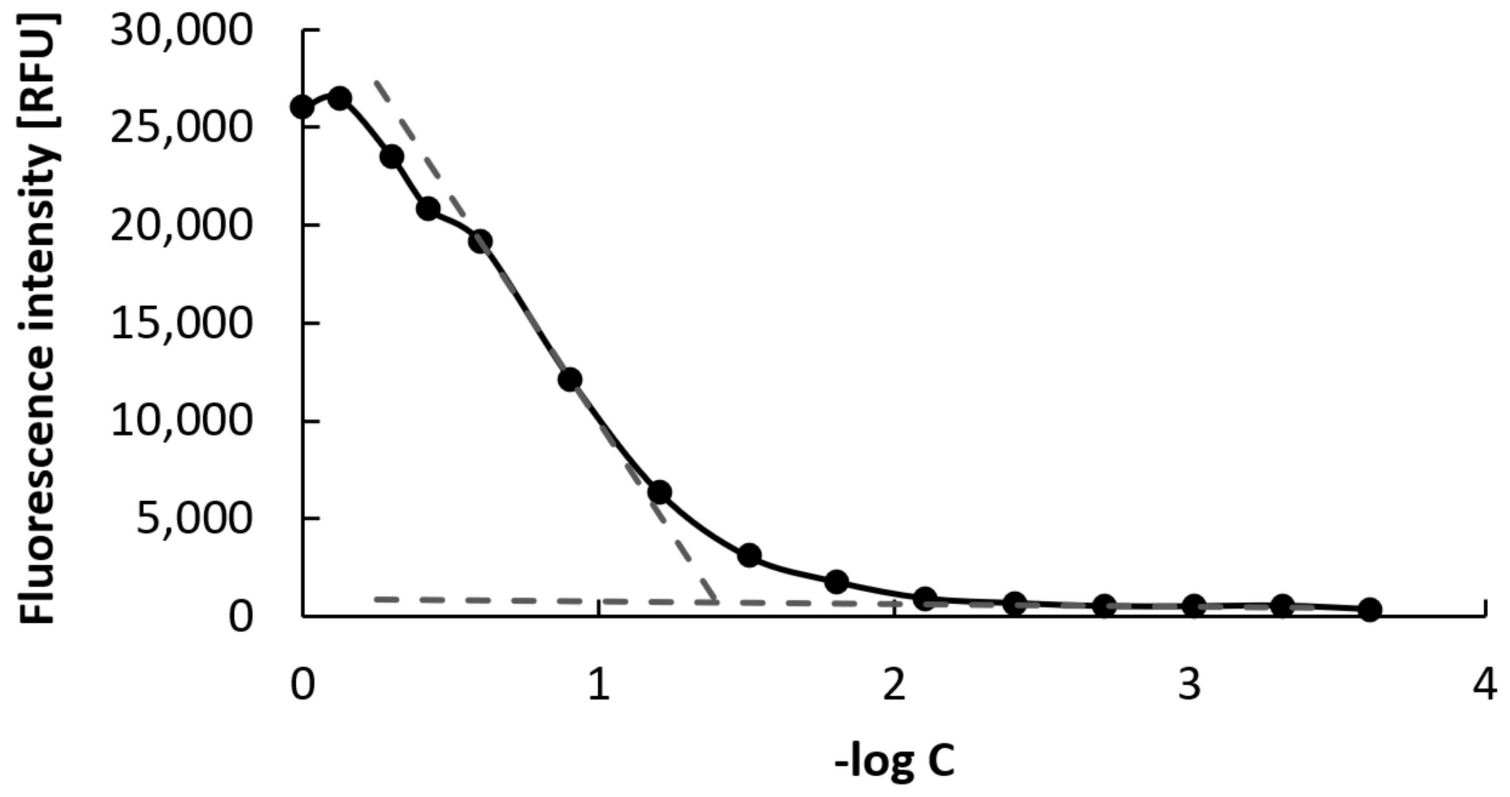
Critical micellar concentration (CMC) of micellar polymer (MP). The dependence of fluorescent intensity on the negative logarithm of the sample concentration for determination of CMC by fluorescence. Nile Red dye in phosphate-buffered saline (PBS; 10 mM, pH 7.4) was used at the concentration of 1 µM (λ_ex_ = 550 nm, λ_em_ = 630 nm).

For CMC determination, the copolymer MP was labeled with NR, which has no fluorescence in a hydrophilic environment, but the fluorescence is detectable in a hydrophobic environment, i.e., in the micellar hydrophobic core. The HPMA-based polymer carriers bearing covalently attached cholesterol moieties spontaneously self-assembled into micelles via direct dissolution in an aqueous solution [36,38]. Importantly, we obtained a comparable value of CMC for MP (0.04 mg·mL^−1^) (Figure 1).

Therefore, we can assume that the micelles containing either CuD or Dox would be stable during the transport to tumor tissue as their CMC is very low and, thus, their enhanced accumulation due to the EPR effect should be expected. After the tumor accumulation and consequent penetration of conjugates to a deeper part of the tumor, we suppose that the micelles should disassemble into single polymer chains (unimers), as their concentration would drop below the CMC. Lastly, we expect faster clearance of linear polymers from the body by renal filtration, which should result in a lower tumor uptake when compared to micellar carriers.

### 4.2. In Vitro CuD Release

**Figure 2:**
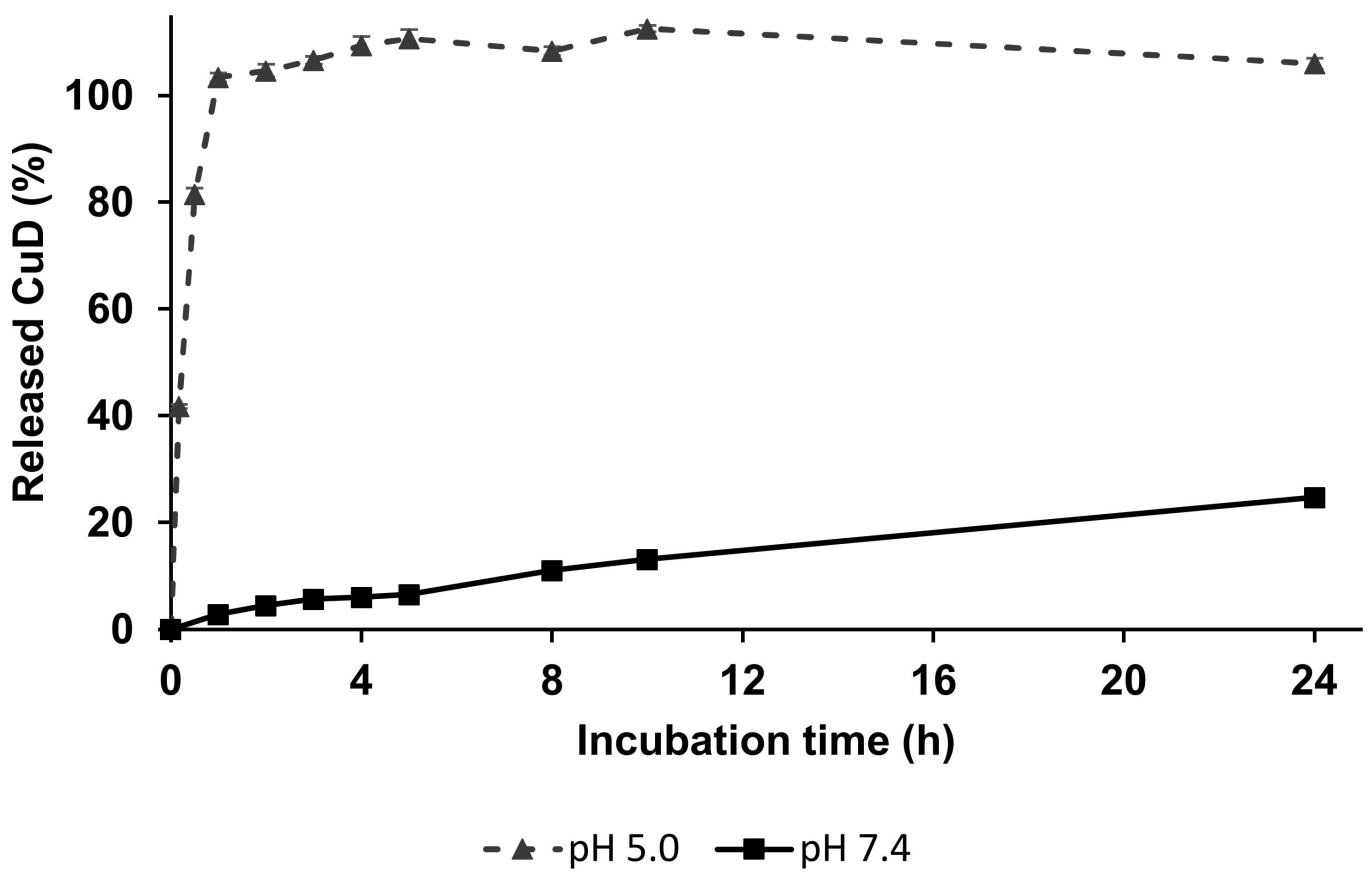
Release of CuD from LP-CuD incubated in 0.1 M phosphate buffer (containing 0.05 M NaCl) at pH 5.0 and pH 7.4 at 37 °C. At indicated time periods, released CuD was determined by HPLC after extraction into chloroform. Values are the mean ± SD (*n* = 3).

The pH-dependent drug release, documented in Figure 2, was enabled by attaching CuD to the polymer carrier employing the hydrazone bond. Results of determination of the spontaneous hydrolysis showed that the hydrazone bond was stable in typical physiological conditions of blood circulation (25% of CuD was released within 24 h incubation at pH 7.4 and 37 °C) and hydrolyzed rapidly in acidic conditions mimicking the lysosome environment of the tumor cells. At pH 5.0 and 37 °C, 42% or 81% of CuD was released within 10 or 30 min, respectively, followed by a release of 100% within 1 h. Representative chromatograms after 8 h of the CuD release are shown in the Supplementary Materials, Section 3, Figure S5. Therefore, LP-CuD fulfills the basic criteria for an efficient anticancer prodrug, that is, stability during blood transportation and fast release of the active drug after entering tumor tissue or cells.

We expect the same pH-sensitive profile also in the case of MP-CuD according to our previous results on similar cholesterol-based micelle-forming polymer conjugates bearing a drug bound via the pH-sensitive hydrazone bond [35,36]. Nevertheless, according to the significant hydrophobic nature of CuD, we assume that partial entrapment of CuD in the hydrophobic micellar core could occur, as recently described for other micellar HPMA-based systems [48]. Such hydrophobic entrapment was found to deplete the anticancer efficacy of the amphiphilic polymer nanomedicines.

### 4.3. In Vitro Cytotoxicity

Both linear and micellar conjugates were characterized by a significant in vitro toxicity toward several murine cancer cell lines (Table 2). As already seen in many types of HPMA-based conjugates carrying various cytotoxic drugs, the free parent drug CuD was more toxic than the relevant conjugates, being characterized by approximately six to seven times lower IC_50_ value than the polymer conjugates. In contrast to small molecules such as free drugs which can enter the cells mainly via diffusion, the internalization of macromolecules into the cells is mainly driven by endocytosis and pinocytosis, which are much slower processes. As a result, a higher drug amount is required within a given time interval to produce a similar cytotoxic effect in vitro compared to the free low-molecular-weight drug. Therefore, polymer conjugates with anticancer drugs are generally less toxic than the free drugs in vitro. This behavior reflects the release of CuD from the polymer carrier. The conjugates should be taken up by the cells and then release the drug from the polymer carrier inside the cells. Moreover, some proportion of the transported drug can be released from the polymer spontaneously outside of the cells, even at typical physiological pH. Presumably, the internalization into the cells and the effect of the free drug occur faster than that of the conjugate. We did not find significant differences between cytotoxicity of the linear and micellar polymer–CuD conjugates. We believe that this could be ascribed to the similar rate of hydrolysis of the hydrazone bond in both polymer conjugates regardless of the type of polymer carrier.

The human permanent cell lines were selected because of their declared overexpression of STAT3 transcription factor, which is frequently documented in many human tumors of various origins. Here, SC-OV-3, OVCAR-3, and DU145 cell lines were explored (Table 3). The rather high CuD toxicity reflected in the lower IC_50_ values in these cell lines is in good agreement with the hypothesis that CuD could act as a STAT3 inhibitor [19,49]. On the other hand, additional mechanisms of toxicity are probably besides the STAT3 inhibition.

**Figure 3:**
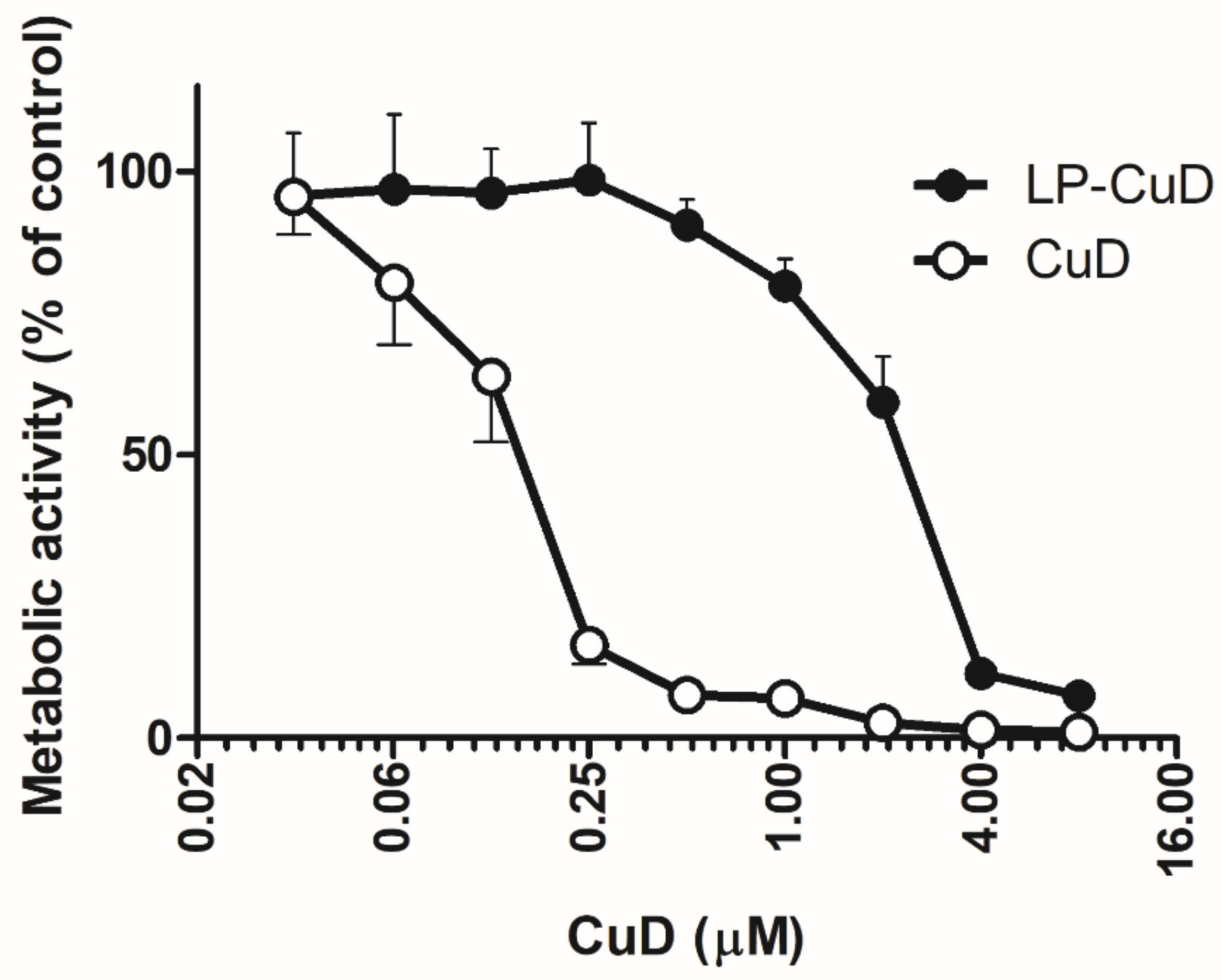
In vitro cytotoxic activity of LP-CuD and free CuD in mitogen-activated murine spleen cells. The cells were isolated from spleens of normal BALB/c mice, plated at 100,000 cells/well in 96-well plates, and activated by 5 mg·mL^−1^ concanavalin A. Concurrently, the LP-CuD and the parent free drug were added at a series of concentrations. Overall metabolic activity of the cells was determined using the standard MTT method after 72 h of cultivation and is expressed as a percentage of the control value (cells with concanavalin A). The IC_50_ values were calculated for the polymer conjugate and the free drug, equal to 2.39 µM CuD equivalent and 0.16 µM CuD, respectively. Four parallel samples per condition were used.

Cytostatic and cytotoxic activity of CuD conjugates was also checked in normal murine spleen cells. Not surprisingly, extensively proliferating spleen cells from normal mice, upon stimulation by a T-cell mitogen (concanavalin A), were found to be quite sensitive to the free parent drug CuD. On the other hand, the cells showed considerable durability to the cytotoxic effect of the LP-CuD conjugate (Figure 3).

### 4.4. In Vivo Activity

The prominent hydrophobic nature of CuD and related inadequate bioavailability restrict its potential therapeutic application. Several reports have shown the anticancer activity of CuD in various models [19,50,51,52], using repeated intraperitoneal (i.p.) or intratumoral (i.t.) administration of the drug with organic solvent as a vehicle. In our hands, i.t. administration of CuD in 4T1-carcinoma-bearing mice led to the development of severe superficial lesions lined with an edge formed by growing tumor cells and no survival prolongation (see Supplementary Materials, Section 4, Figure S6B). Moreover, the higher dosing scheme (6 × 2 mg/kg i.t. every other day) also led to the signs of systemic toxicity (see Supplementary Materials, Section 4, Figure S6A). Therefore, the free parent drug was not included in any further experiment as a control.

Previously, it was thoroughly documented that polymer conjugates carrying various drugs circulate for a significantly extended period of time in peripheral blood as compared with the free parent drug [24,53,54]. Intravenous (i.v.) administration of the LP-CuD conjugate at three dosing schemes did not induce any detectable systemic toxicity (see Supplementary Materials, Section 4, Figure S6C). Thus, the finding proved the hypothesis that CuD transported by the polymer delivery system is applicable and reduces the systemic toxicity of the drug.

**Figure 4:**
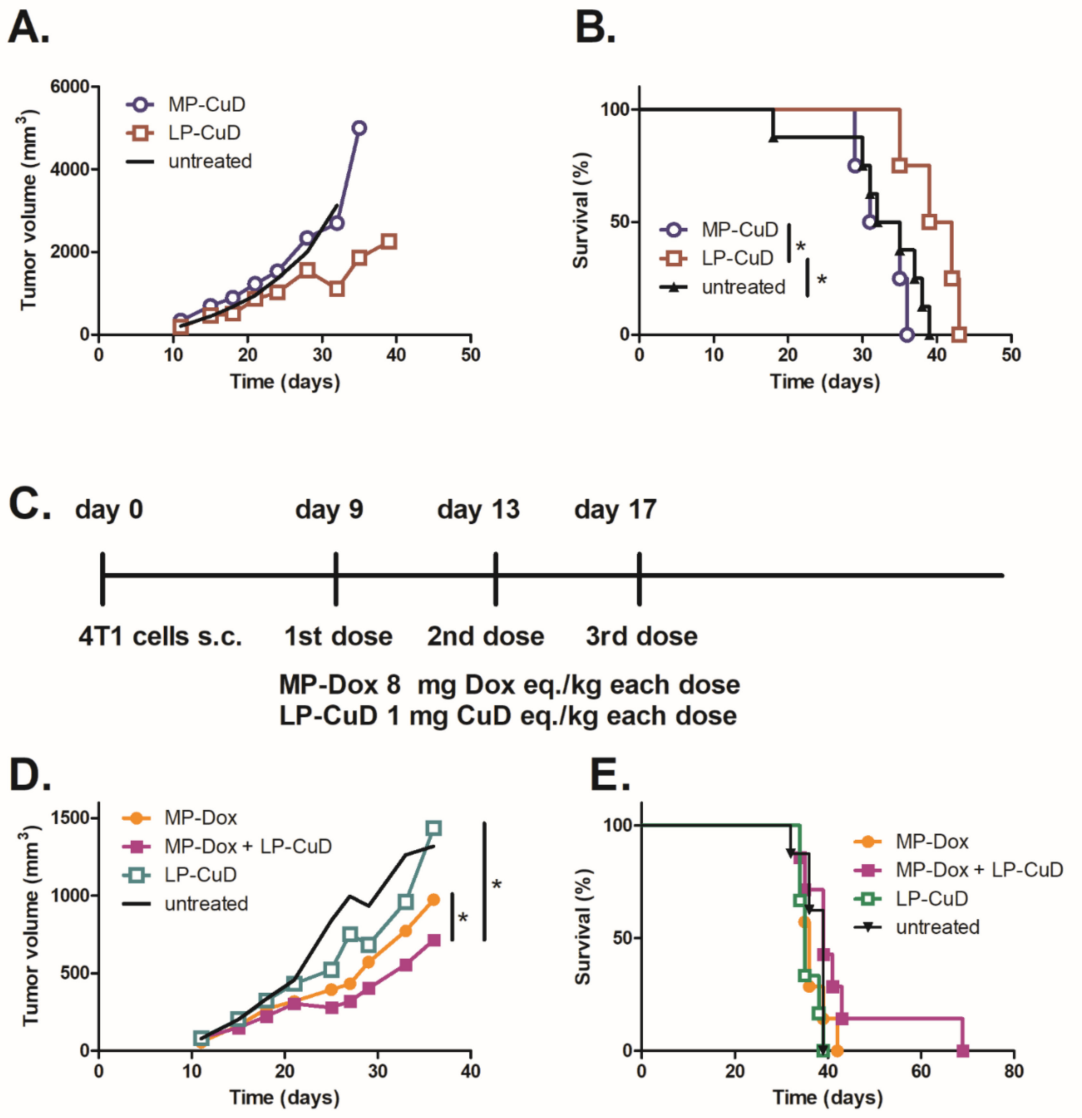
In vivo antitumor effect of the CuD-containing conjugates. The BALB/c mice were transplanted with 2 × 10^5^ 4T1 cells subcutaneously (s.c.) at day 0 and treated after the tumors developed to a measurable size. MP-CuD and LP-CuD were injected intravenously (i.v.) in three partial doses on days 8, 11, and 14, with each dose equivalent to 2.5 mg CuD/kg. Tumor growth (**A**) and survival (**B**) were monitored. No signs of systemic toxicity were recorded; *n* = 4 per condition. In the combination treatment, the mice were treated according to the dosing scheme (**C**). Tumor growth (**D**) and survival (**E**) are depicted. No systemic toxicity was recorded; *n* = 7 per group. Statistical significance: * *p* < 0.05.

Antitumor activity of both LP-CuD and MP-CuD in the in vivo model of 4T1 carcinoma was not very observable, being higher for LP-CuD when applied as a sole treatment (Figure 4A). Specifically, MP-CuD did not reduce tumor growth and also had no impact on the survival of the mice (Figure 4B). We hypothesize that the key issue in such a low therapeutic efficacy lies in the CuD profile release from MP-CuD. As the released CuD should be entrapped in the micelle core and is not liberated from the conjugate, the therapeutic efficacy is reduced. Importantly, the effect of the LP-CuD conjugate was apparent at later stages of the tumor growth and also mildly prolonged the mice survival (Figure 4B). However, the LP-CuD conjugate improved the in vivo activity of micellar conjugate containing Dox (MP-Dox), as evidenced by a reduction in tumor growth (Figure 4D). The dosing scheme (Figure 4C) was chosen so that both the conjugates were below their maximum tolerated dose (MTD) to prevent any systemic toxicity. No significant decrease in body weight was observed (Supplementary Materials, Figure S7). This combination treatment prolonged survival time in one out of the eight animals in the group, whilst the other treatment modalities did not extend the survival time at all (Figure 4E). However, the survival prolongation is a parameter more closely related to the potential clinical applicability. Even with the awareness of this, the results are suggestive of some therapeutic efficacy of the combinational treatment, which should be thoroughly studied in subsequent preclinical testing. The proper and optimized dosing of the combinational immuno-oncotherapy is a major task for the next investigation.

## 5. Conclusions

Both linear and micellar HPMA-based copolymers and their respective conjugates bearing CuD were prepared aiming to solubilize a hydrophobic drug, thus improving its applicability and enabling an effective targeting to solid tumors by means of the EPR effect. The introduction of a cholesterol substituent into the structure of the polymer carrier led to self-assembly of the carrier system into the micellar supramolecular structures. Polymer–drug conjugates were prepared via the reaction of the hydrazide groups of linear and micellar carriers based on HPMA with the keto group from carbon 3 of CuD, providing pH-sensitive drug delivery systems through the use of the hydrazone bond. The conjugates were found to be stable during blood circulation, and a rapid CuD release in the acidic tumor environment was confirmed. Therefore, the conjugates fulfill the pH-dependent release requirement for an efficient anticancer prodrug. Moreover, the in vitro cytostatic/cytotoxic activity tested in several cancer cell lines proved that, after binding to the polymer carrier, whether linear or micellar, the functional capacity of the drug was retained. In vivo, administration of the conjugates showed significantly reduced toxicity as compared with the free parent drug and even the possibility to inject the conjugates intravenously, which is in strong contrast to the devastating effect of the free CuD. The treatment of mice with 4T1 mammary carcinoma, which is a rapidly metastasizing cancer model with a significant impact of immunosuppressive mechanisms, showed the potential of the CuD-containing polymer conjugates in combinatorial treatment together with targeted cytotoxic drugs.

## Figures and Tables

**Table 1:** Physicochemical characteristics of hydrophilic or amphiphilic copolymer precursors (LP or MP, respectively) and their conjugates with CuD (LP-CuD and MP-CuD) or Dox (MP-Dox).

Compound	Content of Hydrazide (mol.%) ^a^	Content of Hydrophobic Moiety (mol.%) ^b^	Content of Drug (wt.%) ^c^	*M*_n_ (g·mol^−1^) ^d^	*M*_w_ (g·mol^−1^) ^d^	*Ð* ^d^	*D*_h_ ± SD ^e^ (nm)
**LP**	4.0	-	-	25,100	26,500	1.05	8.8 ± 0.2
**LP-CuD**	-	-	6.2	27,000	36,200	1.34	12.4 ± 1.0
**MP**	5.0	2.1	-	21,500	26,400	1.23	19.8 ± 0.4
**MP-CuD**	-	2.1	6.7	25,700	39,000	1.51	32.2 ± 1.6
**MP-Dox ^f^**	-	2.0	8.1	14,200	25,500	1.80	29.6 ± 1.0

^a^ The content of hydrazide groups was determined by ultraviolet–visible light (UV–Vis) spectrophotometry using a modified 2,4,6-trinitrobenzene-1-sulfonic acid (TNBSA) assay. ^b^ The content of hydrophobic moieties cholesterol and cholest-4-en-3-one was determined by ^1^H-NMR (600.2 MHz) in dimethyl sulfoxide (DMSO)-*d*_6_. ^c^ The amount of CuD was determined by ^1^H-NMR (600.2 MHz) in DMSO-*d*_6_. The amount of Dox was determined by UV–Vis spectrophotometry at 488 nm in water. ^d^ The number-average molecular weight (*M*_n_), weight-average molecular weight (*M*_w_), and dispersity (*Ð*) of polymer precursors and polymer–drug conjugates were determined using size-exclusion chromatography (SEC) with multiangle light scattering (MALS) and refractive index (RI) detection. The mobile phase consisted of 80% methanol and 20% 0.3 M sodium acetate buffer, pH 6.5. A combination of TSKgel3000AW + TSKgel4000AW columns was used. ^e^ Hydrodynamic diameter (*D*_h_) was evaluated in water (0.5 mg·mL^−1^) using a Nano-ZS instrument, Malvern. Values were not extrapolated to a zero concentration. ^f^ The polymer precursor MP-Dox was not MP, but a copolymer containing hydrazide groups and cholest-4-en-3-one bound via hydrazone bond prepared using free-radical polymerization according to [36].

**Table 2:** Cytostatic/cytotoxic activity of the polymer conjugates carrying CuD in selected murine tumor cells.

	CT26 ^a^	4T1 ^a^	EL4 ^b^
MP-CuD	0.386 ± 0.037 ^c^	0.293 ± 0.033	0.380 ± 0.189
LP-CuD	0.521 ± 0.226	0.512 ± 0.258	0.175 ± 0.002
CuD	0.125 ± 0.020	0.175 ± 0.025	0.048 ± 0.013

^a^ Cytostatic activity of the conjugates and the free drug was determined by ^3^H-thymidine incorporation. ^b^ Cytotoxic activity was determined using the 3-(4,5-dimethylthiazol-2-yl)-2,5-diphenyltetrazolium bromide (MTT) method. ^c^ Mean half maximal inhibitory concentration (IC_50_) value ± SD expressed as equivalent of CuD (µM).

**Table 3:** Cytostatic activity of the polymer conjugates carrying CuD in selected human tumor cells.

	SK-OV-3	OVCAR-3	DU145
MP-CuD	0.185 ± 0.037 ^a^	0.277 ± 0.099	0.517 ± 0.155
LP-CuD	0.183 ± 0.009	0.190 ± 0.057	0.272 ± 0.057
CuD	0.025 ± 0.007	0.053 ± 0.014	0.062 ± 0.022

^a^ Mean IC_50_ value ± SD expressed as equivalent of CuD (µM). Cytostatic activity of the conjugates and the free drug was determined by ^3^H-thymidine incorporation.

## Data Availability

Data sharing not applicable.

## Supplementary Materials

The following are available online at https://www.mdpi.com/1999-4923/13/2/179/s1, Figure S1: ^1^H NMR spectrum of micellar precursor MP, Figure S2: ^1^H NMR spectrum of polymer-drug conjugate LP-CuD, Figure S3: ^1^H NMR spectrum of polymer-drug conjugate MP-CuD, Figure S4: ^13^C NMR spectra of CuD and polymer-drug conjugate MP-CuD, Figure S5: Representative chromatograms after 8 h of CuD release, Figure S6: Systemic toxicity and survival of mice treated with free CuD or LP-CuD, Figure S7: Systemic toxicity of the combination treatment with MP-Dox and LP-CuD.
