# Supplementary material for: HPMA-Based Copolymers Carrying STAT3 Inhibitor Cucurbitacin-D as Stimulus-Sensitive Nanomedicines for Oncotherapy
Source: Pharmaceutics. 2021 Jan 28;13(2):179. doi: 10.3390/pharmaceutics13020179 (PMC7911143; doi:10.3390/pharmaceutics13020179)
Supplement: Supplementary file 1 [file pharmaceutics-13-00179-s001.pdf]

pharmaceutics

Supplementary Materials: HPMA-Based Copolymers Carrying STAT3 Inhibitor Cucurbitacin-D as Stimuli-Sensitive Nanomedicines for Oncotherapy

Marina R. Tavares, Klára Hrabánková, Rafał Konefał, Martin Kaňa, Blanka Říhová, Tomáš Etrych, Milada Šírová and Petr Chytil *

1. Synthesis of polymer precursors

1.1. Detailed synthesis of linear precursor LP

HPMA (526.9 mg, 3.68 mmol) and CTA -TTC (5.1 mg, 25 μmol) were dissolved in *t*-butanol (4.86 mL), then mixed with a solution of MA-AH-NHNH-Boc (100.3 mg, 0.32 mmol) and V-70 (3.8 mg, 12.50 μmol) in DMA (0.86 mL) – the mixture contained 0.7 M solution of monomers. The reaction mixture was poured into a glass ampoule, bubbled with argon and sealed. The polymerization was carried out in a thermostat-controlled water bath at 30°C for 72 h and, after cooling the ampoule, the reaction mixture was dropped into a mixture of acetone and diethyl ether (2/1; 150 mL). The precipitate was filtered and purified by reprecipitation from methanol (4.5 mL) into the mixture of acetone and diethyl ether (2/1; 150 mL). After centrifugation at 7800 RPM for 3 minutes, the copolymer was filtered and dried under vacuum (408 mg, 65 %). The trithiocarbonate end group was removed by the addition of AIBN (62 mg) into a solution of copolymer (408 mg) in dry DMA (5 mL). The mixture was poured into a glass ampoule, bubbled with argon and sealed. The reaction was carried out in a thermostat-controlled water bath at 80 °C for 3 h and, after cooling the ampoule, the sample was isolated by dropping the solution into 150 mL of ethylacetate. The precipitate was filtered and purified by reprecipitation from methanol (5 mL) into ethylacetate (150 mL). After centrifugation at 7800 RPM for 3 minutes, the copolymer was filtered and dried under vacuum (384 mg, 94 %). For the Boc group removal, the copolymer was dissolved in Q-H_2O (7 mL), transferred to an ampoule and placed into a thermostat-controlled oil bath at 100 °C for 2 hours. After cooling the ampoule, the solution was freeze-dried to yield the linear copolymer LP with reactive hydrazide groups, as a white powder (353 mg, 92 %).

1.2. Detailed synthesis of micellar precursor MP

A solution of HPMA (500 mg, 3.49 mmol), MA-AH-NHNH-Boc (110.7 mg, 0.35 mmol), MA-AH-cholesterol (44.5 mg, 0.08 mmol), CTA-AIBN (4.8 mg, 22 μmol) and AIBN (3.1 mg, 11 μmol) was prepared in *t*-butanol (4.3 mL) – the mixture contained 0.9M solution of monomers. The reaction mixture was poured into a glass ampoule, bubbled with argon and sealed. The polymerization was carried out in a thermostat-controlled water bath at 70°C for 16 h and, after cooling the ampoule, the reaction mixture was dropped into a mixture of acetone and diethyl ether (2/1; 150 mL). The precipitate was filtered and purified by reprecipitation from methanol (7 mL) into the mixture of acetone and diethyl ether (2/1; 150 mL). After centrifugation at 7800 RPM for 3 minutes, the copolymer was filtered and dried under vacuum (572 mg, 87 %). The dithiobenzoate end group was removed by the addition of AIBN (86 mg) into a solution of copolymer (572 mg) in dry DMA (6 mL). The mixture was poured into a glass ampoule, bubbled with argon and sealed. The reaction was carried out in a thermostat-controlled water bath at 80 °C for 3 h and, after cooling the ampoule, the sample was isolated by dropping the solution into a mixture of acetone and diethyl ether (2/1; 150 mL). The precipitate was filtered and purified by reprecipitation from methanol (6 mL) into the mixture of acetone and diethyl ether (2/1; 150 mL). After centrifugation at 7800 RPM for 3 minutes, the copolymer was filtered and

Publisher's Note: MDPI stays neutral with regard to jurisdictional claims in published maps and institutional affiliations.

Copyright: © 2021 by the author. Licensee MDPI, Basel, Switzerland. This article is an open access article distributed under the terms and conditions of the Creative Commons Attribution (CC BY) license (http://creativecommons.org/licenses/by/4.0/).

dried under vacuum (532 mg, 93 %). For the Boc group removal, the copolymer was dissolved in Q-H_2O (10 mL), transferred to an ampoule and placed into a thermostat-controlled oil bath at 100 °C for 2 hours. After cooling the ampoule, the solution was freeze-dried to yield the micellar copolymer MP, with reactive hydrazide groups and cholesterol substituents, as a white powder (473 mg, 89 %). NMR data for calculation of cholesterol content is shown in the Supplementary Material, Section 2.

2. NMR data

2.1. NMR data of micellar precursor MP

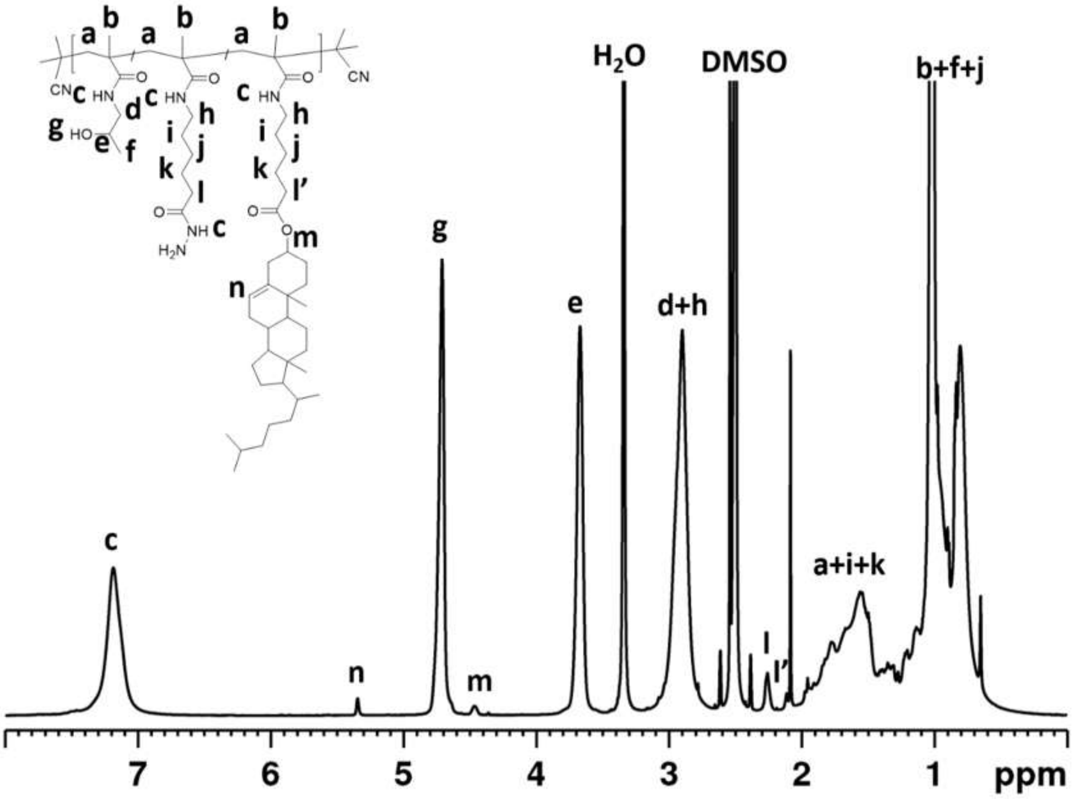

Figure S1. ^{1}H NMR spectrum of micellar precursor MP (600.23 MHz in DMSO-d_6 at 295 K).

2.2. NMR data of polymer conjugates bearing CuD

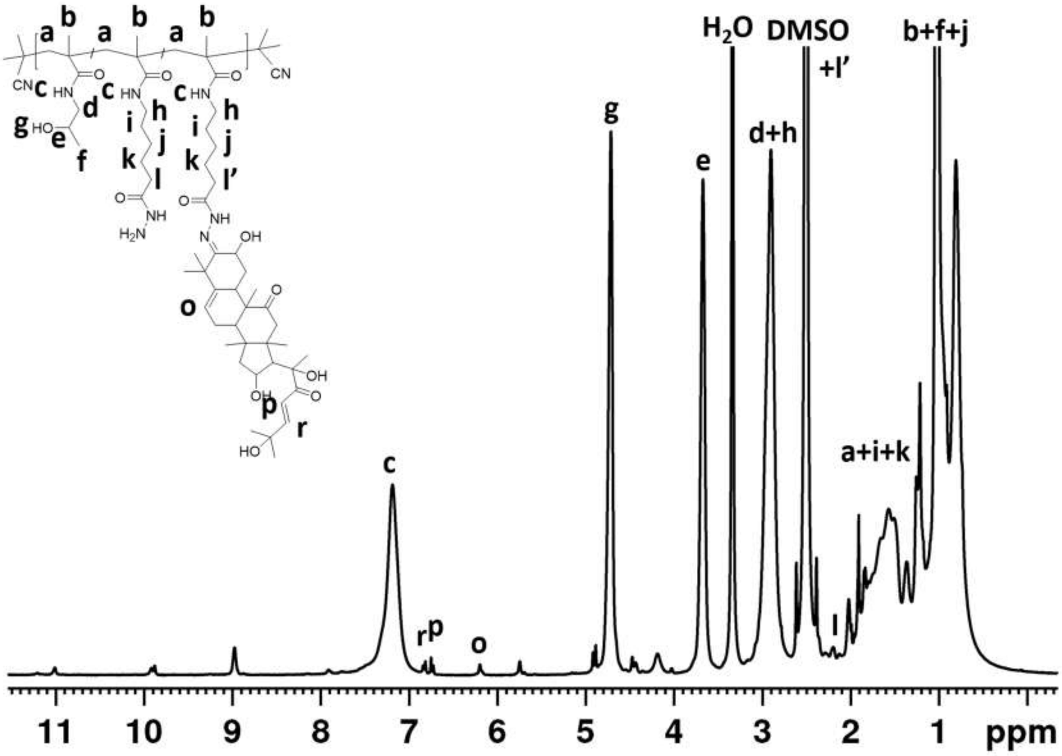

Figure S2. ^{1}H NMR spectrum of polymer-drug conjugate LP-CuD (600.23 MHz in DMSO-d_6, 295 K).

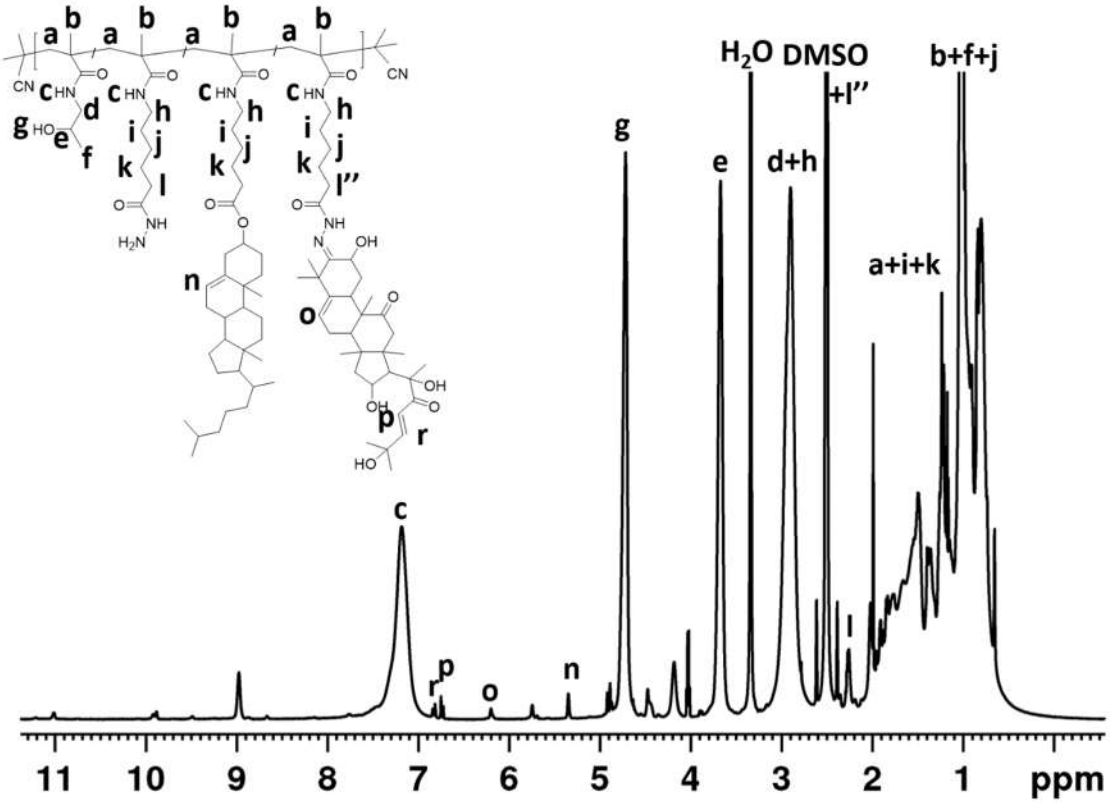

Figure S3. ^{1}H NMR spectrum of polymer-drug conjugate MP-CuD (600.23 MHz in DMSO-d_6 at 295 K).

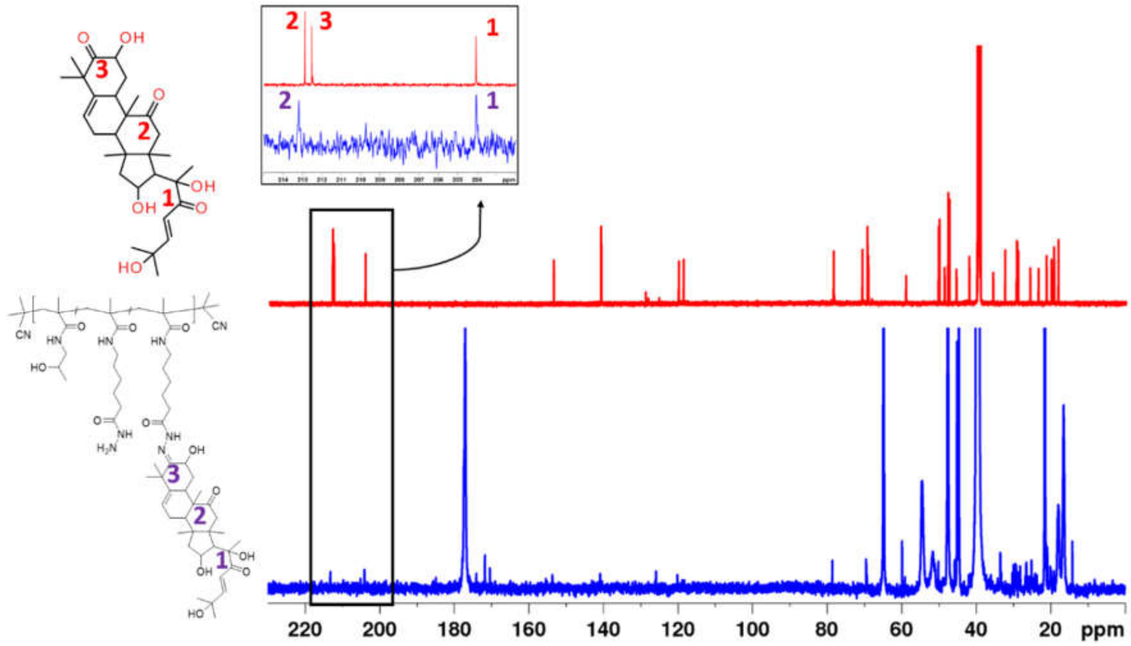

Figure S4. ^{13}C NMR spectra of CuD (red) and polymer-drug conjugate MP-CuD (blue) measured in DMSO-*d*$_6$ at 295 K. Only carbonyl carbons assignments from CuD are marked to show which group is reacted.

3. Drug release representation

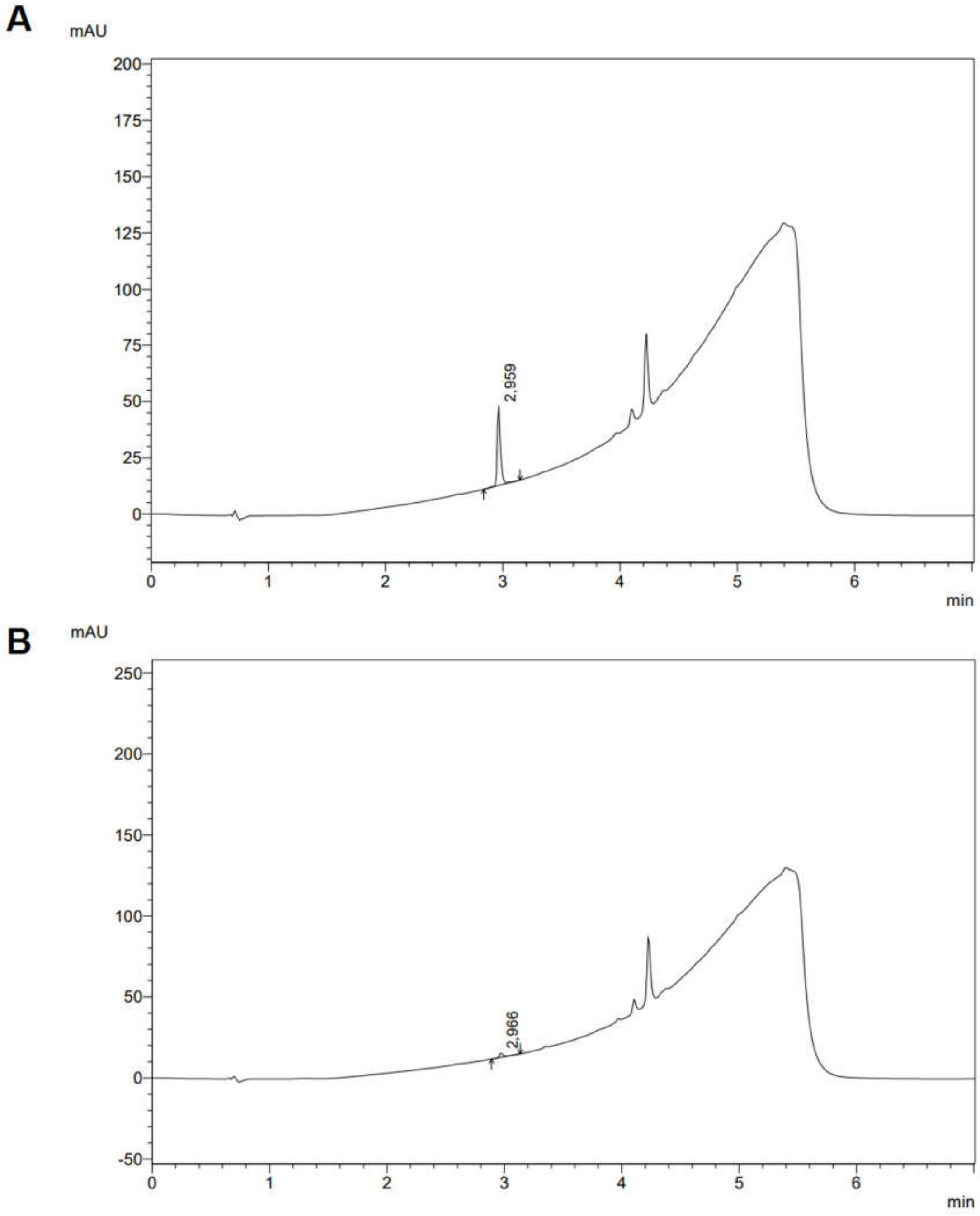

Figure S5. Representative chromatograms after 8 h of CuD release. (A) at pH 5.0 **(B)** at pH 7.4. CuD retention time t_R = 3 min (peaks above t_R = 4 min are artefacts from the HPLC column).

4. *In vivo* evaluation

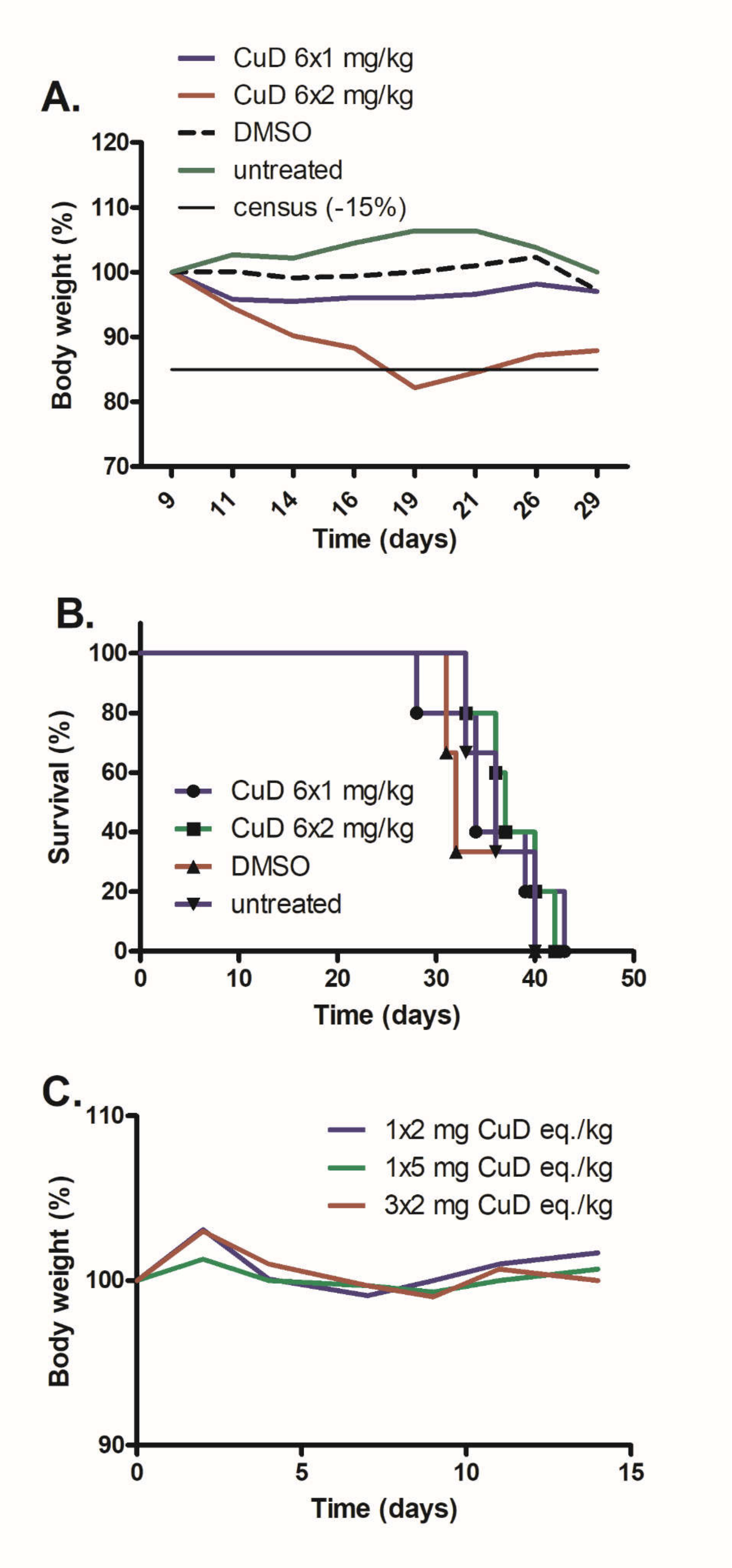

Figure S6. Systemic toxicity and survival of mice treated with free CuD or LP-CuD. The mice with s.c.-transplanted 4T1 tumors (5x10^5 cells at day 0) were treated with 6 consecutive doses of CuD at 1mg/kg or 2 mg/kg each, administered i.t. in two sites within the tumor focus at days 8, 10, 12, 14, 16, and 18 post tumor transplantation. The drug was dissolved in DMSO and further diluted with PBS, volume of each dose was 0.1 mL. Equivalent dose of DMSO was used for control. Tumor growth (not shown), body weight **(A)** and survival of the mice **(B)** was regularly monitored. Normal BALB/c mice were injected i.v. with LP-CuD at three dosage schemes: single dose of the conjugate equivalent of 2 mg CuD/kg, single dose of 5 mg CuD/kg, or three consecutive doses equivalent of 2 mg CuD each, injected at days 0, 3, and 6. The conjugate was dissolved in PBS. Body weight of the mice is shown in **(C)**.

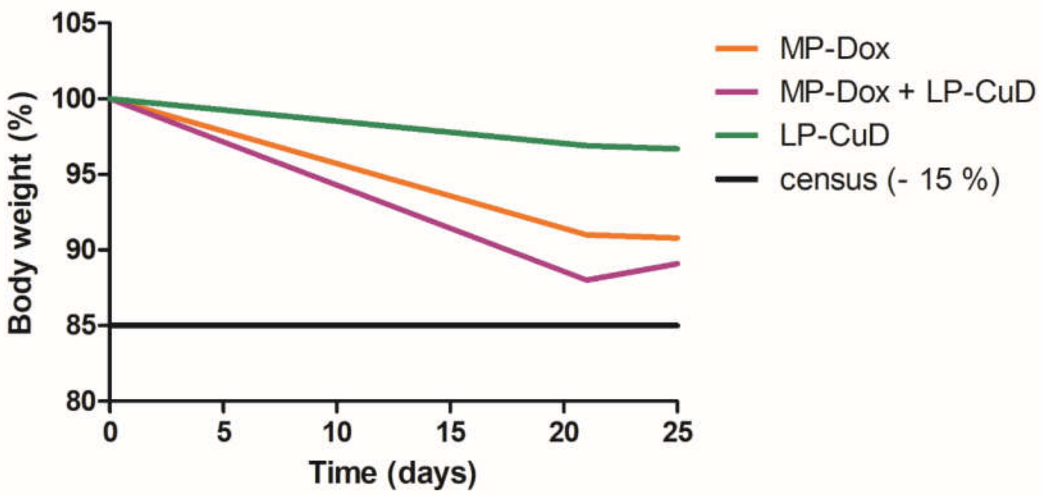

Figure S7. Systemic toxicity of the combination treatment with MP-Dox and LP-CuD. The BALB/c mice were transplanted with 2x10^5 4T1 cells s.c. at day 0, and treated after the tumors developed to a measurable size. MP-Dox and LP-CuD were injected i.v. in three partial doses at days 9, 13, and 17, each dose equivalent of 8 mg Dox /kg and 1 mg CuD/kg. The treatment scheme is depicted in Figure 4 C, main text. Potential systemic toxicity was monitored using the body weight as the measurable parameter; reduction of more than 15 % was considered the cut-off value. n=7 per group.
